# Determining the Critical Period of Continuous Waterlogging in Maize: An Analysis of Physiological, Biochemical, and Transcriptomic Traits

**DOI:** 10.3390/plants15020330

**Published:** 2026-01-21

**Authors:** Denglong Chen, Cong Peng, Zhiming Liu, Wanrong Gu, Fanyun Yao, Lichun Wang, Yujun Cao, Yongjun Wang

**Affiliations:** 1 Institute of Agricultural Resources and Environment, Jilin Academy of Agricultural Sciences (Northeast Agricultural Research Center of China)/Northeast Key Laboratory of Crop Physiology, Ecology and Cultivation, Ministry of Agriculture and Rural Affairs, Changchun 130033, China; 13168224405@163.com (D.C.); pengcong0410@163.com (C.P.); liuzhiming5050@163.com (Z.L.); yaofanyun@163.com (F.Y.); wlc1960@163.com (L.W.); 2 College of Agriculture, Jilin Agricultural University, Changchun 130118, China; 3 College of Agriculture, Northeast Agricultural University, Harbin 150006, China; wanronggu@neau.edu.cn

**Keywords:** maize, waterlogging stress, physiological and biochemical characteristics, transcriptome analysis

## Abstract

Waterlogging stress severely limits crop photosynthesis and energy supplies, resulting in significant yield reductions. However, the critical duration of waterlogging stress during the maize jointing stage remains unclear, and the physiological and molecular mechanisms underlying its effects on photosynthetic efficiency and energy synthesis in maize require further investigation. In this study, we systematically analyzed the responses of physiological traits, transcriptomic profiles, and the yield formation in maize (*Zea mays* L.) to varying waterlogging durations imposed during the jointing stage, including 0 days (CK), 2 days (F2), 4 days (F4), 6 days (F6), 8 days (F8), and 10 days (F10). Our results indicate that the (1) grain weight (GW) showed no significant difference between F2 and CK. However, the GW in F4, F6, F8, and F10 decreased significantly by 17.49%, 26.45%, 60.24%, and 100.00%, respectively, compared to the CK. (2) Compared with the CK, the malondialdehyde content progressively increased from F4 to F10, while antioxidant enzyme activity gradually decreased. The chlorophyll content declined by 29.93% to 57.38%, and net photosynthetic efficiency decreased by 13.82% to 38.93%. Although the leaf sucrose content in from F4 to F10 gradually decreased, the leaf starch content remained stable in F4 and F6. In contrast, the starch content in F8 and F10 leaves was significantly reduced by 37.55% and 47.60%, respectively, compared with CK. (3) A transcriptomic analysis revealed that during from F2 to F4, genes encoding photosystem I subunit protein, such as PSAD, and the cytochrome b6f complex proteingene PETC were downregulated. At F6, these key genes encoding photosynthetic proteins were upregulated. However, at F8 and F10, their expression was significantly downregulated. Concurrently, genes related to ATP synthesis (e.g., ATPD) as well as starch and sucrose metabolism (e.g., SPP2, SS1) were also downregulated. In summary, when waterlogging stress persists for no longer than 6 days, plants can maintain their starch content to supply energy for growth, thereby ensuring basic developmental needs. When waterlogging persists for more than 6 days, energy synthesis is impaired, and the nutrient transport to the grains is significantly inhibited, ultimately resulting in a substantial reduction in yield. Therefore, 6 days of waterlogging can be considered the critical threshold for significant yield loss in maize during the jointing stage.

## 1. Introduction

Maize is one of the world’s most important food crops, playing an indispensable role in ensuring global food security and improving living standards [1]. However, in the context of climate change, the stability of agricultural production faces significant challenges. The unpredictability of global climate change has resulted in more frequent extreme weather events, notably a significant increase in the intensity and frequency of heavy rainfall and flooding. Consequently, flooding has become an escalating problem for global agriculture [2,3,4]. Maize, a crop sensitive to waterlogging, is vulnerable to flooding stress throughout its entire growth cycle. This stress results in a reduced ear length and diameter, an increased ear tip, and average yield losses exceeding 29%, posing a significant threat to stable corn production [5,6,7]. Most existing studies on maize waterlogging stress have focused on the sensitive seedling stage, confirming that waterlogging at this stage inhibits root and shoot growth, reduces root activity, increases malondialdehyde content, induces plant wilting or even death, and disrupts nutrient absorption and distribution, thereby exacerbating growth inhibition [8,9]. Additionally, the research on waterlogging stress at different growth stages has illustrated that waterlogging during the seedling and jointing stages causes the most significant damage to maize growth and yield [10].

In recent years, heavy rainfall events have frequently occurred around the jointing stage (V6) in China’s northeast regions, which are major maize-producing areas, Since the period from jointing to tasseling represents the vegetative growth phase, stress during this stage impairs nutrient accumulation. This deficiency subsequently limits nutrient transport to the kernels during the grain-filling stage, ultimately affecting yield formation. Therefore, the V6 stage represents significant practical and guiding importance in relation to waterlogging stress, rationalizing its selection.

Damage caused by waterlogging stress damage to in maize is related to a complex physiological process, primarily manifested by the inhibition of the photosynthetic system. Under anoxic conditions, plants actively downregulate photosynthetic functions to conserve energy [11]. However, this adaptive strategy leads to an excessive reduction in the photosynthetic electron transport chain, which in turn triggers the overaccumulation of reactive oxygen species (ROS) in chloroplasts. Excessive ROS disrupts the intracellular redox balance, induce membrane lipid peroxidation, and subsequently cause chlorophyll degradation and leaf wilting. These effects directly damage photosynthetic structures and inhibit photosynthetic efficiency [12,13,14]. Meanwhile, waterlogging stress induces stomatal closure in leaves, reducing the supply of carbon dioxide [15,16]. When substrates for carbon assimilation are limited but light energy continues to be absorbed, photorespiration intensifies. This further impedes the normal accumulation of photosynthetic products, ultimately inhibiting plant growth and development [17,18].

A transcriptome analysis indicates that waterlogging stress significantly upregulates or downregulates the expression of numerous genes involved in the energy metabolism and photosynthetic pathways [19]. Among these, the functional stability of core photosynthetic components, such as photosystem I (PSI) and the cytochrome b6f complex (Cytb6f), is crucial for maintaining photosynthetic efficiency under stress conditions. The PSI subunit gene PSAD facilitates electron transport by maintaining the structural integrity of the complex, while the Cytb6f complex gene PETC mediates energy conversion within the photosynthetic electron transport chain [20,21,22]. When photosynthesis is persistently constrained, it impairs the supply of photosynthetic products to the grains, triggering an energy supply crisis [23]. To counteract this energy shortage, plants initiate a series of metabolic remodeling processes. For instance, hypoxia induced by waterlogging increases the activity of transaminases and hexokinase in roots, reflecting heightened energy demands and enhanced glycolytic activity [24]. Plants induce amylase to decompose stored starch into utilizable glucose and adjust the mode of sucrose utilization to prioritize energy mobilization for basal metabolism [25,26]. This shift in energy metabolism from efficient aerobic respiration to less efficient glycolysis is a common survival strategy employed by plants under hypoxic stress. However, this inevitably leads to a reduction in the supply of carbohydrates and ATP, causing a significant upregulation of starch synthase genes (homologs of SS1/GBSS), which promotes the rapid breakdown of starch in leaves to provide substrates for glycolysis [27]. Meanwhile, the elevated expression accelerates sucrose-6-phosphate dephosphorylation, increasing the flux of transportable sucrose to maintain the root energy supply and cellular osmotic balance [28]. However, the sustained downregulation of genes involved in energy metabolism may impair the translocation of photosynthates to the ears, ultimately limiting yield formation [29,30].

While previous studies have characterized variations in the key physiological processes of maize under waterlogging stress, common changes include reduced photosynthetic efficiency and impaired nutrient transport. However, there is a conspicuous lack of systematic, in-depth investigations into the physiological and molecular regulatory mechanisms governing maize responses to different waterlogging durations at the jointing stage, especially for core metabolic functions such as photosynthetic efficiency and energy metabolism. Therefore, this study used “Fumin 985”, a major maize variety promoted in Jilin Province, China, as the experimental material to systematically analyze the effects of different waterlogging durations (0, 2, 4, 6, 8, and 10 days) on maize yield, physiological characteristics, and transcriptomic responses. The study aims are as follows: (1) elucidate the differential expression patterns of genes related to photosynthesis and energy synthesis in maize under varying durations of waterlogging stress; (2) determine the threshold duration of waterlogging stress for maize, providing a scientific basis and practical guidance for stress management in agricultural production.

## 2. Results

### 2.1. Grain Yield per Plant and Its Components of Maize Under Waterlogging Stress

Under the 2-day waterlogging (F2) treatment, the grain weight (GW) demonstrated no significant difference compared to the control (CK). However, as the waterlogging duration increased, the GW decreased significantly. Treatments F4, F6, and F8 exhibited significant decreases of 17.49%, 26.45%, and 60.24% (*p* < 0.01), respectively, compared to CK. At 10 days of waterlogging (F10), the maize ear development was severely inhibited, resulting in complete crop failure. Regarding the constituent factors, the ear length in the F2 and F4 treatments demonstrated no significant difference compared to the control (CK), whereas the F6 and F8 treatments exhibited significantly increased ear lengths. The ear kernel number and 100-grain weight exhibited trends similar to the GW performance, both decreasing with prolonged waterlogging durations. Compared to CK, the F4–F8 treatments showed declines of 10.00% to 44.44% (*p* < 0.01) in the ear kernel number and 6.26% to 27.45% (*p* < 0.05) in the 100-grain weight (Table 1).

### 2.2. Effects of Waterlogging Stress on Physiological Indicators in Maize Leaves

**Figure 1:**
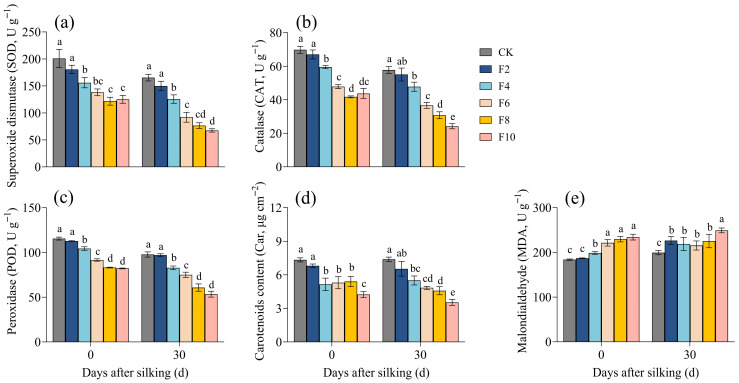
Changes in the antioxidant system and malondialdehyde content in maize leaves under waterlogging stress. (**a**) Superoxide dismutase (SOD) activity; (**b**) Catalase (CAT) activity; (**c**) Peroxidase (POD) activity; (**d**) Carotenoid content; (**e**) Malondialdehyde (MDA) content. Different lowercase letters above the bars indicate significant differences among treatments at *p* < 0.05.

In both periods all other inundation regimes significantly affected protective enzyme activities, except for the 2-day inundation treatment (F2), which showed no difference from the CK (Figure 1a–c). However, superoxide dismutase (SOD), catalase (CAT), and peroxidase (POD) activities in F4–F10 exhibited a continuous decline with extended inundation durations, falling by 23.10–47.35%, 15.92–46.73%, and 12.23–36.42% (*p* < 0.01), respectively, relative to CK. The carotenoid content (Car) in waterlogging treatments decreased as the number of inundation days increased, with more pronounced differences observed during longer durations (Figure 1d). Conversely, the malondialdehyde (MDA) content, which reflects the membrane lipid peroxidation, gradually increased with prolonged waterlogging, showing significant increases of 8.98%, 13.98%, 18.72%, and 26.12% (*p* < 0.05) in F4–F10 compared to CK.

### 2.3. Relevance Analysis

**Figure 2:**
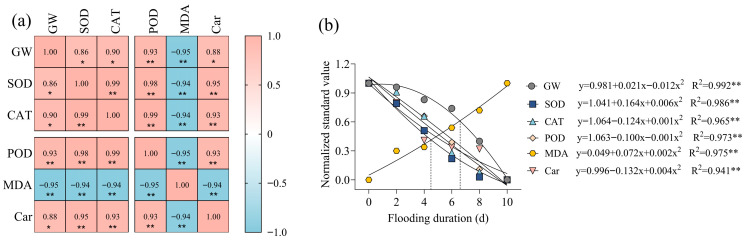
Correlation analysis among various indicators and normalized fitting analysis. (**a**) Correlation matrix of different indicators obtained by Pearson correlation analysis: GW represents grain weight; SOD (superoxide dismutase), CAT (catalase), POD (peroxidase), MDA (malondialdehyde content), and Car (carotenoid content). The color intensity and value in the matrix represent the correlation coefficient (red for positive correlation, blue for negative correlation). (**b**) Normalized fitting curves of each indicator with waterlogging duration days, with corresponding fitting equations and R^2^ values marked. * and ** indicate significant differences at the levels of *p* < 0.05 and *p* < 0.01, respectively.

A correlation analysis revealed that SOD, CAT, POD, and Car exhibited highly significant positive correlations with GW, whereas MDA demonstrated highly significant negative correlations with GW, SOD, CAT, POD, and Car. Following the normalization of these physiological indicators and after fitting their variation curves to the GW, we identified 6 days of waterlogging as the critical threshold for maize (Figure 2). To clarify differences across the critical period, the waterlogging process was split into two phases: the initial 6 days of waterlogging stress and days 8–10 post-stress initiation, which supports further in-depth investigations.

### 2.4. Differentially Expressed Gene Screening and Analysis

**Figure 3:**
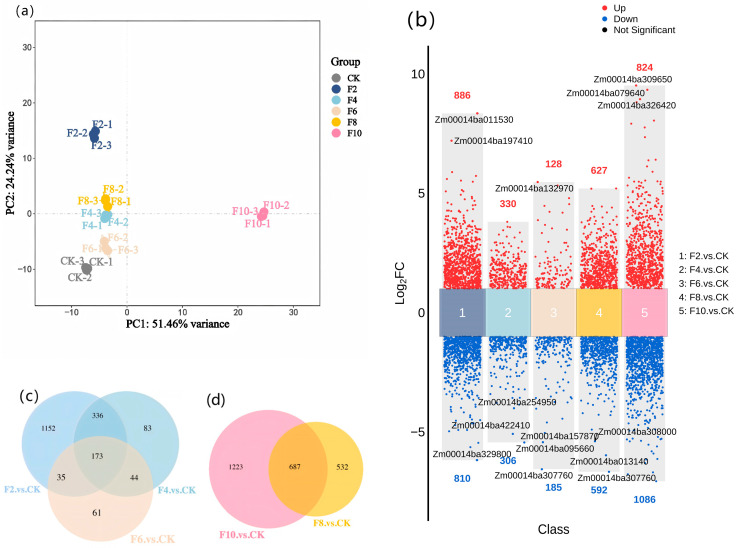
Transcriptome analysis. (**a**) Principal component analysis of the samples. (**b**) Volcano plot of DEGs. (**c**,**d**): Venn diagrams of DEGs under short-term and long-term waterlogging stress, respectively.

To assess the variation between CK, F2, F4, F6, F8, and F10, we performed a principal component analysis (PCA). The results showed that the first principal component explained 51.46% of the variance, while the second principal component contributed 24.24%, collectively explaining 75.70% of the total variance (Figure 3a). Samples from each waterlogging stress treatment group clustered were closely clustered, indicating low intragroup variation and high reliability.

To gain deeper insights into the response mechanisms of plants to varying durations of waterlogging, we conducted a differential gene expression analysis. This analysis revealed a phased response of maize to waterlogging stress. During the 2–6 d waterlogging period, the numbers of both upregulated and downregulated differentially expressed genes (DEGs) showed a decreasing trend, with a total of 173 common DEGs identified. In contrast, the number of DEGs increased significantly during the 8–10 d period, reaching a total of 687 common DEGs. Notably, in the F10 treatment, the number of downregulated DEGs was significantly higher than the number of upregulated DEGs (Figure 3b–d).

### 2.5. Functional Enrichment Analysis Using GO and KEGG

**Figure 4:**
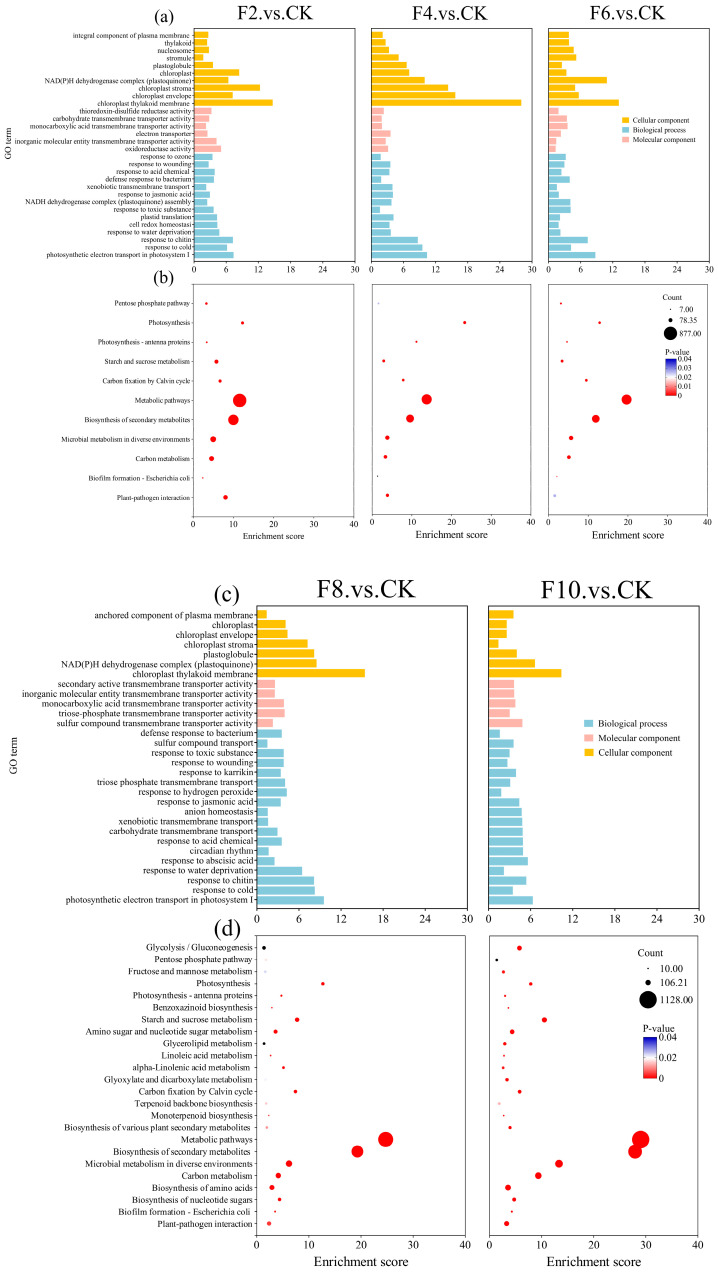
The GO enrichment of DEGs in the biological process and KEGG pathway enrichment. (**a**,**c**) Gene Ontology (GO) enrichment analysis of DEGs in F2 vs. CK, F4 vs. CK, F6 vs. CK, F8 vs. CK, and F10 vs. CK comparisons; (**b**,**d**) Kyoto Encyclopedia of Genes and Genomes (KEGG) pathway enrichment analysis of DEGs in F2 vs. CK, F4 vs. CK, F6 vs. CK, F8 vs. CK, and F10 vs. CK comparisons.

An analysis of the top 30 significantly enriched Gene Ontology (GO) terms revealed that F2–F6 were collectively enriched in cellular components such as the chloroplast thylakoid membrane, chloroplast envelope, chloroplast stroma, and thylakoid, all of which are closely associated with photosynthetic processes. Similarly, F8–F10 were enriched in photosynthesis-related pathways (Figure 4a–c).

The KEGG analysis further elucidated the functions of DEGs (Figure 4b–d). During 2 to 6 days of waterlogging stress, the core enriched pathways were primarily associated with energy synthesis, including photosynthesis, carbon metabolism, and starch and sucrose metabolism. However, after 8 to 10 days, the enriched pathways expanded significantly beyond photosynthesis to encompass broader metabolic domains, such as glycan metabolism, lipid metabolism, and amino acid biosynthesis.

### 2.6. Analysis of Metabolic Pathways and Related Genes

**Figure 5:**
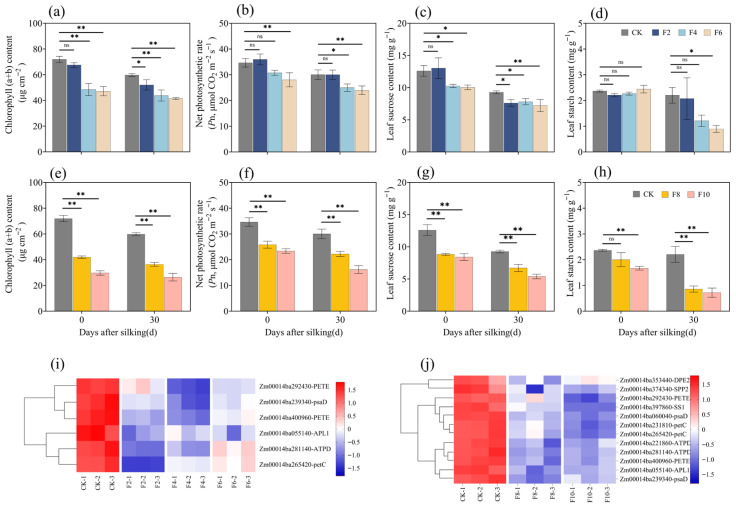
Analysis of differentially expressed genes (DEGs) under waterlogging stress. Panels (**a**–**d**) and (**f**–**i**) represent the net photosynthetic rate and the contents of chlorophyll (a + b), sucrose, and starch under short-term and long-term waterlogging stress, respectively. Panels (**e**,**j**) show clustering heatmaps of the expression levels of DEGs related to photosynthesis and sucrose and energy metabolism under short-term and long-term waterlogging stress, respectively. * and ** indicate significant differences at the levels of *p* < 0.05 and *p* < 0.01, respectively. ns indicates no significant difference (*p* > 0.05).

Measurements conducted after varying durations of waterlogging demonstrated that at F2, the chlorophyll (a + b) content (Chl) and net photosynthetic rate (*P*n) did not differ significantly from the CK. However, after 4 days of waterlogging, both parameters exhibited a significant decline. Specifically, the Chl in the CK group was significantly higher than that in the F4–F10 groups by 29.93%, 32.71%, 40.52%, and 57.38% (*p* < 0.01), respectively. The *P*n was significantly higher by 13.82%, 19.58%, 25.68%, and 38.93% (*p* < 0.01) (Figure 5a,b,f,g). In terms of energy synthesis, the sucrose content in F4–F10 decreased significantly by 17.29%, 21.04%, 28.82%, and 36.78% (*p* < 0.01), respectively, compared to the CK (Figure 5c,h). During the 2–6 d waterlogging period, their leaf starch content did not differ from that of the CK. However, the leaf starch content in F8 and F10 decreased significantly by 37.55% and 47.60% (*p* < 0.01), respectively, compared to the CK (Figure 5d,i). These physiological findings further confirm that photosynthesis and energy metabolism are the primary pathways through which maize responds to waterlogging stress.

A heatmap analysis of genes related to photosynthesis and starch-sucrose metabolism revealed that under conditions F2 to F4, most photosynthesis-related proteins (PSAD, PETC, ATPD, and PETE) and some energy synthesis proteins (APL1) exhibited a downregulation trend, whereas F6 exhibited upregulation. In F8–F10, photosynthesis-related genes remained downregulated, and energy synthesis proteins (DPE2, SPP2, and SS1) also exhibited significant downregulation, except for APL1 (Figure 5e,j).

### 2.7. qRT-PCR Validation of Differentially Expressed Genes

**Figure 6:**
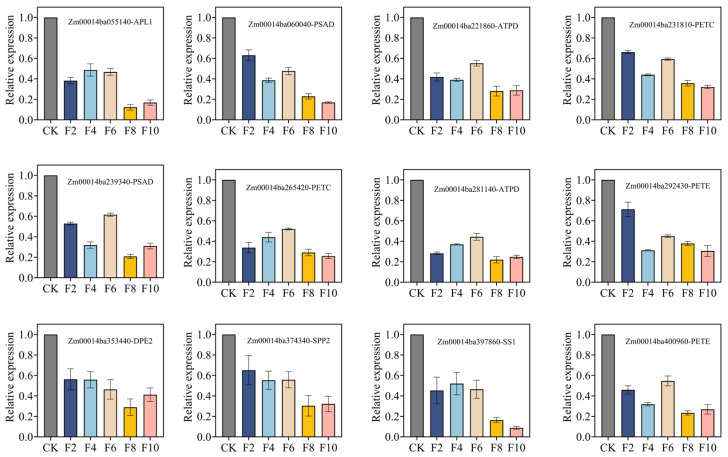
Relative expression levels of related genes under waterlogging stress. The relative expression of the target gene was calculated using the 2^−ΔΔCt^ method.

The qRT-PCR analysis of the expression levels of 12 key genes in response to waterlogging stress revealed (Figure 6) that all genes were generally downregulated following inundation regimes compared to the 0-day control.

## 3. Discussion

Maize yield formation is determined by two key processes: the fixation of assimilates through photosynthesis and the efficient translocation and partitioning of these assimilates to the grains, which is fueled by energy synthesis. Reduced photosynthesis directly limits nutrient synthesis in maize kernels, thereby hindering yield formation [9]. Previous studies have confirmed that the impact of waterlogging stress on the yield varies significantly across different growth stages, with the jointing stage (V6) experiencing a greater reduction in yield than the tasseling stage (VT). This occurs because the photosynthetic system sustains more severe damage at the V6 stage, resulting in a significant decrease in dry matter accumulation and, ultimately, reduced yield [10]. The results of this study are consistent with these findings. As the duration of the waterlogging stress increased (from 4 to 10 days), the reduction in grain weight (GW) progressively intensified, with decreases ranging from 17.49% to 100.00% (Table 1).

Photosynthesis is a fundamental physiological process in plants and one of the most sensitive to abiotic stress. Research indicates that waterlogging stress reduces photosynthetic rates, ultimately inhibiting plant growth and development [9]. Furthermore, when stress induces an excessive accumulation of reactive oxygen species, leading to membrane lipid peroxidation and disruption of membrane homeostasis, plant photosynthesis is similarly impaired. This effect is directly evidenced by a sharp increase in the malondialdehyde (MDA) content [13,31,32]. The dynamic changes in antioxidant enzyme activities (SOD, CAT, and POD) observed in this study reflect the plant’s efforts to mitigate ROS-induced damage. However, the gradual decline in these protective components with prolonged waterlogging indicates a breakdown of the antioxidant defense system. Beyond the 6-day waterlogging threshold, the sharp decrease in antioxidant capacity coincides with substantial reductions in the chlorophyll content and net photosynthetic rate (Figure 5), suggesting that the photosynthetic apparatus shifts from reversible damage to irreversible impairment. This threshold-dependent transition provides a physiological explanation for the nonlinear relationship between the waterlogging duration and yield loss.

The plant’s response to water stress is a complex regulatory process. At the molecular level, plants respond through the extensive reprogramming of the transcriptome and the induction of numerous stress-response genes. These genes encode protective metabolites that facilitate plant survival and growth during stress [33,34,35]. Notably, ethylene response factor VII (ERFVII) family transcription factors are well-documented core regulators of plant hypoxic stress responses [16,23], with ZmEREB180 identified as a key member mediating maize waterlogging tolerance in previous studies [36]. The ERFVII proteins are known to coordinate the expression of downstream genes involved in anaerobic respiration, ROS scavenging, and energy metabolism remodeling, which are critical for plant adaptation to waterlogging-induced hypoxia [23]. Although the present study focused on photosynthetic and energy metabolism pathways rather than specific transcription factor regulation, the physiological and molecular changes observed (e.g., ROS accumulation, photosynthetic system damage, and energy metabolism disorder) are consistent with the downstream effects of ERFVII-mediated signaling. This suggests that the two-phase regulatory pattern of maize under waterlogging stress (conservative adaptation within 6 days, severe damage beyond 6 days) may be indirectly modulated by ERFVII family genes such as ZmEREB180, which provides a broader molecular context for interpreting our findings. The dynamic changes in differentially expressed genes (DEGs) observed in this study with a reduction in DEG numbers during 2–6 days of waterlogging and a the dramatic increase during 8–10 days reflect a two-phase regulatory pattern. The initial decrease in DEGs may represent a conservative adaptive response, whereas the subsequent surge, particularly the predominance of downregulated genes at 10 days, indicates widespread transcriptional repression associated with severe stress damage. The GO functional enrichment analysis further revealed that DEGs from both the 2–6 and 8–10 d were significantly enriched in cellular components closely associated with photosynthesis, including thylakoid membranes, chloroplast envelopes, and chloroplast stroma. The KEGG analysis revealed that during the 2–6 d, DEGs were primarily enriched in energy synthesis-related pathways, including photosynthesis, carbon metabolism, and starch/sucrose metabolism. By the 8–10 d period, the enrichment expanded to broader metabolic domains, such as sugar metabolism, lipid metabolism, and amino acid synthesis (Figure 4). Our findings also demonstrated that the expression levels of key photosynthetic genes, PSAD and PETC, exhibited a downward trend during days 2 to 4, with a brief upregulation at F6, followed by sustained downregulation from F8 to F10 (Figure 5). Multiple studies have demonstrated that PSAD helps maintain the structural integrity of the photosystem I (PSI) complex through interactions with core PSI subunits (such as PSAA and PSAB) and peripheral proteins (such as PSAE), playing a crucial role in electron transfer and structural stability [37]. Additionally, its interaction with PSAA and the chloroplast-encoded YCF3 protein is crucial for the assembly of the photosystem I complex [38,39]. As one of the four major subunits of Cytb6f, PETC performs specific electron transfer functions essential for normal photosynthesis. When the PETC expression is downregulated, the electron transfer efficiency of the Cytb6f complex decreases directly, hindering the timely transfer of electrons generated by photosystem II (PSII) to photosystem I (PSI). This inhibition reduces the overall photosynthetic efficiency [20]. Consequently, when continuous waterlogging exceeds 6 days, core photosystem genes are significantly downregulated, impairing the function of the encoded photosynthetic complexes and diminishing electron transfer efficiency, ultimately resulting in a substantial decline in photosynthetic productivity.

Although photosynthesis decreased during 6 days of waterlogging, there was no significant difference in the leaf starch content compared to the control (Figure 5d). The maintaining of the leaf starch content plays a crucial role in abiotic stresses such as waterlogging and drought. During stress periods, starch serves as a buffer for carbon and energy. Beyond its function as an energy storage molecule, starch also acts as a precursor for osmotic regulation, directly contributing to plant stress resistance [40,41]. This indicates that when photosynthesis is limited, sugar metabolism can stabilize cell membranes and protoplasts, thereby enhancing osmotic resistance to stress and providing energy and carbon sources [42,43]. However, our results demonstrate that at 8 and 10 days of waterlogging, although photosynthetic rates declined, the leaf starch content decreased significantly by 37.55% and 47.60%, respectively, compared to the control. ATP synthesis-related genes, such as ATPD, along with carbohydrate synthesis genes like SPP2 and SS1, were significantly downregulated after more than 6 days of waterlogging (Figure 5j). This suggests that when plants experience waterlogging-induced hypoxia, the sustained downregulation of genes involved in carbohydrate mobilization and anaerobic fermentation impairs the ATP production necessary to maintain substrate levels, thereby compromising growth [44]. Our results demonstrate that when waterlogging stress exceeds a threshold duration of approximately 6 days (5.86 days), the synthesis of storage compounds is concurrently suppressed, leading to insufficient energy reserves. Consequently, plants lack adequate nutrients to support grain development, ultimately resulting in significant yield reductions or even complete crop failure. The critical threshold of yield loss caused by 6-day waterlogging identified in this study aligns with the previously reported threshold range of 4 to 6 days in maize research [45]. These studies similarly indicate that waterlogging lasting longer than 6 days results in an irreversible decline in photosynthetic efficiency.

In this study, Fumin 985, a locally dominant maize cultivar, was selected for waterlogging stress experiments to ensure that the conclusions drawn could directly inform regional agricultural production practices. The identified 6-day critical waterlogging threshold, along with the associated physiological responses and molecular regulatory patterns, may vary across different maize genotypes. Future research will expand the range of tested cultivars to verify the generalizability of these findings and to explore the genetic basis underlying differences in waterlogging tolerance across diverse maize varieties.

## 4. Materials and Methods

### 4.1. Experimental Materials and Design

The experiment was conducted from May to October 2024 at the Gongzhuling Campus of the Jilin Academy of Agricultural Sciences, Jilin Province, China (43°53′ N, 124°81′ E). The maize waterlogging experiment was carried out in a custom-made PVC box measuring 220 cm in length, 110 cm in width, and 60 cm in height. The experiment simulated field planting conditions, with two rows of maize planted in the box at a row spacing of 65 cm and a plant spacing of 22 cm, with 10 plants per row. The soil is classified as silty clay loam (Mollisols, according to USDA Soil Taxonomy). Soil samples were collected from the 0–30 cm layer of farmland, thoroughly mixed, and sieved to remove impurities before being placed into containers. Each container was filled with a 10 cm layer of gravel at the bottom to serve as a filter layer, after which the soil was added and fully waterlogging to enable settling. The soil surface was maintained 5 cm below the container rim to prevent runoff during flooding. The soil nutrient content included organic matter at 18.4 g kg^−1^, total nitrogen at 1.57 g kg^−1^, available phosphorus at 37.6 mg kg^−1^, and available potassium at 135.3 mg kg^−1^. The pH was 6.7, and the soil field capacity was 27.6%.

The maize variety tested was ‘Fumin 985’, the primary variety promoted in Jilin Province, China. ‘Fumin 985’ is a commercial hybrid maize cultivar widely cultivated in the study area, which exhibits good adaptability and high yield potential locally. This cultivar was selected to ensure that the research results could directly guide regional agricultural production practices. Fertilizer application rates were 240 kg hm^−2^ of nitrogen fertilizer (N), 100 kg hm^−2^ of phosphorus fertilizer (P_2_O_5_), and 120 kg hm^−2^ of potassium fertilizer (K_2_O). The fertilizer was used a controlled-release maize compound with an N:P_2_O_5_:K_2_O ratio of 24:10:12 and was applied in a single pre-planting application, following standard production practices. The trial sowing date was 10 May, and the harvest date was 3 October. Waterlogging treatments began at the maize jointing stage (V6), with each treatment subjected to continuous waterlogging for 0 (CK), 2 (F2), 4 (F4), 6 (F6), 8 (F8), and 10 days (F10). Water-logging stress was imposed on the maize cultivar ‘Fumin 985’ at the V6 growth stage (jointing stage). For plants exposed to 0–6 d of water-logging, the duration from the termination of stress to sample collection at the silking stage was 28 days. For plants with 8 d of water-logging, this interval reached 30 days. Given that no ears developed under the 10 d water-logging treatment, the sampling time point for all experimental groups was standardized to the 30-day interval associated with the 8 d water-logging treatment. During waterlogging, water was maintained uniformly at a depth of 3–5 cm on the soil surface. After the inundation regimes were completed, drainage was performed using manual pumping and a drain valve at the bottom of the box was maintained until all standing water was removed from the soil. After the completion of the waterlogging stress treatment, all agronomic management measures for the waterlogged maize plots were standardized to match those implemented in the control group.

### 4.2. Photosynthetic Pigments and Net Photosynthetic Rate Measurement

At the silking stage and 30 days after silking (DAS), a fresh portion of the ear leaves was selected from each plot. Ten holes, each 6 mm in diameter, were punched from the leaves. The leaf samples were then immersed in 15 mL of 95% ethanol and kept in the dark for 48 h. Using a Shimadzu UV-2600 spectrophotometer (Shimadzu Co., Kyoto, Japan), the absorbance of the solution was measured at wavelengths of 665 nm, 649 nm, and 470 nm. The relevant calculation method is as follows [46]:

$$\begin{array}{l} \mathrm{Chl}\;a=13.95{\mathrm{OD}}_{665}-6.88{\mathrm{OD}}_{649}\\ \mathrm{Chl}\;b=24.96{\mathrm{OD}}_{649}-7.32{\mathrm{OD}}_{665}\\ \mathrm{Car}=(1000{\mathrm{OD}}_{470}-2.05\mathrm{Chl}\;a-114.8\mathrm{Chl}\;b)/245\\ \mathrm{Car}\;\mathrm{content}\;(\mathrm{\mu g}\;{\mathrm{cm}}^{-2})=\mathrm{Car}\times 15/2.826\\ \mathrm{Total}\;\mathrm{chlorophyll}\;\mathrm{content}\;(\mathrm{\mu g}\;{\mathrm{cm}}^{-2})=(\mathrm{Chl}\;a+\mathrm{Chl}\;b)\times 15/2.826 \end{array}$$

OD_665_, OD_649_, and OD_470_ represent the relative optical densities at wavelengths of 665, 649, and 470 nm, respectively; Chla, Chlb, and Car denote the concentrations of chlorophyll a, chlorophyll b, and carotenoids (mg L^−1^); where 15 is the extraction volume (mL) and 2.826 is the sample area (cm^2^). During the same time period, net photosynthetic rate (*P*n) of ear leaves was measured using a portable photosynthesis system (Li 6400, Li-Cor Biosciences, Lincoln, NE, USA) on sunny days between 9:00 and 11:00 a.m.

### 4.3. Leaf Sucrose and Starch Content

At 0 and 30 DAS, ear leaves were collected. The leaf sucrose content was determined using the phenol-sulfuric acid method, while the leaf starch content was measured using the anthrone colorimetric method.

### 4.4. Protective Enzyme Activity and Malondialdehyde (MDA) Content

At 0 and 30 DAS, ear leaves were sampled. A 0.5 g aliquot of leaf tissue, stored at −80 °C, was thoroughly ground in liquid nitrogen, followed by the addition of 5 mL of phosphate buffer (pH was 7.8). After complete extraction, the mixture was centrifuged at 4 °C and 10,000 rpm for 20 min. The supernatant was collected as the enzyme solution for analysis. The superoxide dismutase (SOD) activity was determined using the nitroblue tetrazolium (NBT) method; the peroxidase (POD) activity was measured via the guaiacol colorimetric method; the catalase (CAT) activity was via the ultraviolet absorption method; and the malondialdehyde (MDA) content was determined using the thiobarbituric acid (TBA) method [47].

### 4.5. Grain Yield per Plant and Its Components

At the physiological maturity stage (R6), all ears from each treatment were harvested, and traits such as the kernel rows per ear, kernels per row, barren tip length, 100-kernel weight, and grain weight per ear were measured.

### 4.6. Transcriptome Sequencing and Analysis

Ear leaves were sampled at the maize silking stage (R1) for total RNA extraction. The total RNA quality was assessed as follows: the concentration and purity were determined using a NanoDrop spectrophotometer (Thermo Scientific NanoDrop 2000, Thermo Scientific, Waltham, MA, USA), and integrity was verified by RNA-specific agarose gel electrophoresis or an Agilent 2100 Bioanalyzer with the RNA 6000 Nano Kit (Cat. No. 5067-1511, Agilent Technologies Inc., Santa Clara, CA, USA). Raw image data generated from sequencing were converted to sequence data (raw reads) via base calling. The cDNA library was sequenced on the Illumina HiSeq platform to obtain raw sequencing files in FASTQ format, and the quality of the sequencing data from 18 samples was evaluated using FastQC (Available online: https://www.bioinformatics.babraham.ac.uk/projects/fastqc/; accessed on 15 October 2025). Raw reads were subjected to quality control with Trimmomatic software (Available online: http://www.usadellab.org/cms/index.php?page=trimmomatic; accessed on 15 October 2025) to remove adapter-containing and low-quality reads, yielding high-quality clean reads.

The gene IDs used in this study strictly adhere to the standard annotation format of the maize reference genome ZmB73_RefGen_v4 (NCBI accession: GCF_000005005.2), with a unified prefix “Zm000014daXXXXXX” that is consistent with the official annotation information of GCF_000005005.2. The complete genome sequence and corresponding annotation files (e.g., genomic.gff.gz, feature_table.txt.gz) of ZmB73_RefGen_v4 are publicly available from the NCBI repository at https://ftp.ncbi.nlm.nih.gov/genomes/all/GCF/000/005/005/GCF_000005005.2_B73_RefGen_v4/ (accessed on 15 October 2025).

DEGs among different gene sets were analyzed using DESeq software (v1.38.3), with input data being the gene count matrix annotated based on B73_V4, which serve as the input data. Gene expression differences between samples were calculated using the FPKM (Fragments Per Kilobase of transcript per Million mapped reads) method. The expression ratio of waterlogging stress vs. the control (normal) was defined as the fold change, with screening criteria set at a *p*-value < 0.05 and |log_2_FoldChange| > 1. The GO (Gene Ontology) enrichment analysis was performed using topGO software (v2.50.0), where *p*-values were calculated via the hypergeometric distribution method (significance threshold: *p*-value < 0.05) to identify GO terms significantly enriched in DEGs (all/upregulated/downregulated), thereby clarifying the main biological functions of DEGs. The GO annotation information was synchronized with the corresponding annotation of B73_V4 in the MaizeGDB database to ensure consistency with the gene ID annotation. The KEGG (Kyoto Encyclopedia of Genes and Genomes) pathway enrichment analysis was conducted using clusterProfiler software (v4.6.0).

### 4.7. qRT-PCR Validation of Differentially Expressed Genes (DEGs)

The expression patterns of differential genes in leaves were detected by a quantitative real-time PCR (qRT-PCR). Total RNA was extracted using the Trizol method. A total of 500 ng of DNase-treated total RNA was reverse-transcribed with the PrimeScript™ RT Reagent Kit with gDNA Eraser (Takara, Japan). Primers for target genes were designed using the online tool Primer-BLAST (https://www.ncbi.nlm.nih.gov/tools/primer-blast/; accessed on 15 October 2025). Zm4cfin2 was used as the reference gene. All primers were synthesized by Shanghai Biotech Bioengineering Co., Ltd., Shanghai, China, and their sequences are provided in Table 2. The qRT-PCR assays were performed on an ABI QuantStudio 6 Real-Time PCR System (Thermo Fisher Scientific, Waltham, MA, USA) using the SYBR^®^ Green Premix Pro Taq HS qPCR Kit II (Accurate Biology, Guangzhou, China). Each reaction was conducted in a 20 μL volume containing 1 μL of cDNA as the template. The qRT-PCR program was set as follows: initial denaturation at 96 °C for 10 min, followed by 35 cycles of denaturation at 96 °C for 30 s, annealing at 60 °C for 30 s, and extension at 72 °C for 30 s, with a final extension at 72 °C for 10 min and holding at 4 °C indefinitely. Three independent biological replicates were performed for each treatment. The relative expression levels of target genes were calculated using the 2^−ΔΔCt^ method. The expression trends of target genes obtained by the qRT-PCR were compared with the log_2_FoldChange expression trends calculated based on FPKM in transcriptome sequencing. The results demonstrated that the expression change trends (upregulation/downregulation) of all detected genes were consistent with those of RNA-seq, verifying the reliability of the transcriptome sequencing data.

### 4.8. Statistical Analyses

The data are presented as the mean values of three biological replicates for each treatment. Data were analyzed using one-way analysis of variance (ANOVA) followed by Tukey’s post hoc test. Significance thresholds were set at *p* < 0.05 and *p* < 0.01. Principal component analysis (PCA), correlation analysis, and ANOVA were performed using SPSS version 27.0 (IBM Corp., Armonk, NY, USA; www.ibm.com/spss). Graphs and tables were generated with GraphPad Prism 10.0 (GraphPad Software, San Diego, CA, USA; www.graphpad.com).

## 5. Conclusions

**Figure 7:**
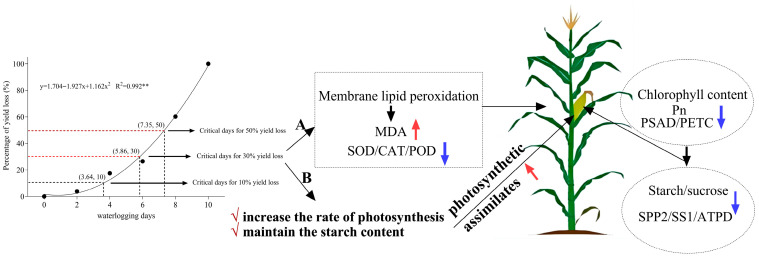
The grain weight loss under different waterlogging durations. The critical threshold (5.86 days, rounded to 6 days for clarity) was calculated via the quadratic regression fitting of the relationship between waterlogging days and the yield loss percentage. ** *p* < 0.01 (Pearson correlation analysis). When the waterlogging duration exceeds this threshold (A), it induces leaf membrane lipid peroxidation (elevated MDA, reduced SOD/CAT/POD), which in turn decreases the chlorophyll content, net photosynthetic rate (*P*n), and expression of photosynthetic proteins (PSAD/PETC), as well as reducing the leaf starch/sucrose content and expression of related proteins (SPP2/SS1/ATPD). These effects impair the assimilate transport to ears, leading to >30% per-plant yield loss. (B) Recommendations: Implement cultivation measures to enhance the photosynthetic rate and maintain leaf starch contents, thereby promoting assimilate transport to grains. In the figure, black arrows = processes/relationships; blue arrows = decreases; red arrows = increases; and checkmarks = recommended measures.

The results of this study indicate that approximately 6 days of waterlogging (5.86 days) is the critical threshold during the maize jointing stage (Figure 7). Before this period, maize can maintain its leaf starch content and sustain nutrient transport to the grain by activating antioxidant defense systems, even under conditions of reduced photosynthetic efficiency. After this threshold, membrane lipid peroxidation intensifies, leading to a comprehensive downregulation of photosynthetic genes such as PSAD and PETC. Consequently, the photosynthetic system collapses, failing to support the efficient transport of sucrose and starch to the grain. Simultaneously, the downregulation of energy metabolism genes (ATPD, SPP2, and SS1) impairs assimilate transport, ultimately causing a substantial decline in yield.

## Figures and Tables

**Table 1:** Productivity per plant and its component factors in maize.

Treatments	Bare Tip Length (cm)	Kernel Numbers per Ear	100-Kernel Weight (g)	Grain Weight (g ear^−1^)
CK	0.58 ± 0.02 c	560.33 ± 14.13 a	34.53 ± 0.48 a	189.94 ± 0.48 a
F2	0.62 ± 0.08 c	545.67 ± 6.06 a	34.14 ± 0.63 a	182.72 ± 0.63 a
F4	0.93 ± 0.03 c	504.30 ± 18.62 b	32.37 ± 0.19 b	156.72 ± 0.19 b
F6	2.79 ± 0.24 b	464.59 ± 9.06 c	29.61 ± 0.06 c	139.71 ± 0.06 c
F8	4.20 ± 0.33 a	311.30 ± 7.14 d	25.05 ± 0.01 d	75.52 ± 0.01 d
F10	-	-	-	-

Note: Different lowercase letters following the data in the same column indicate significant differences (*p* < 0.05) between different waterlogging durations. A dash indicates that the panicles were empty and shriveled on the 10th day of waterlogging. Mean ± SD, where the mean represents the average value of 3 replicates, and SD denotes the standard deviation.

**Table 2:** Primer sequences of candidate reference genes.

Gene Name	Primer Name	Primer Sequence (5′→3′)	Tm °C
Zm00014ba239340-PSAD	Zm00014ba239340-PSAD-qF	TCTTCCCCAACGGCGAGGTG	60
	Zm00014ba239340-PSAD-qR	TGCTCTTGCCGGTGAACTTGAC	60
Zm00014ba055140-APL1	Zm00014ba055140-APL1-qF	CGTTCTCGGATAGGCTCCACTGTG	60
	Zm00014ba055140-APL1-qR	CTCTCCTCTTTCCACGGCAGTTTC	60
Zm00014ba281140-ATPD	Zm00014ba281140-ATPD-qF	GCCAAGAACGTCCGCCTCAAG	60
	Zm00014ba281140-ATPD-qR	ACGCTCATGTCGATCAAGTTGGAG	60
Zm00014ba221860-ATPD	Zm00014ba221860-ATPD-qF	GCCCAGAACGTCCGCATCAAG	60
	Zm00014ba221860-ATPD-qR	GTCGATTAAGCTGGAGCCGTCAC	60
Zm00014ba265420-PETC	Zm00014ba265420-PETC-qF	GGGCTTGGCTTGAGCATAGGC	60
	Zm00014ba265420-PETC-qR	CCGAGGAGGAGGAGGTTCATCAG	60
Zm00014ba231810-PETC	Zm00014ba231810-PETC-qF	GCAAGCCAGCGGAGCATCAC	60
	Zm00014ba231810-PETC-qR	GCCCAGGAGGAGGAGGTTCATC	60
Zm00014ba060040-PSAD	Zm00014ba060040-PSAD-qF	CCAAGGAGCAGGTGTTCGAGATG	60
	Zm00014ba060040-PSAD-qR	TGATCTTGTACTTGGAGCGCAACC	60
Zm00014ba397860-SS1	Zm00014ba397860-SS1-qF	GCTCTTGCTGCTCGTGGTCAC	60
	Zm00014ba397860-SS1-qR	GCCAAAGCATGGAATCCGAATGTG	60
Zm00014ba292430-PETE	Zm00014ba292430-PETE-qF	CCCGCACAACGTCGTCTTCG	60
	Zm00014ba292430-PETE-qR	CGGTGAGGGTGACGGAGTAGG	60
Zm00014ba374340-SPP2	Zm00014ba374340-SPP2-qF	GGAGGCAATGGTTCCTGATGATGG	60
	Zm00014ba374340-SPP2-qR	CGCTGTTCTGTCTCTGGCTGAAG	60
Zm00014ba353440-DPE2	Zm00014ba353440-DPE2-qF	AGGACCTTGGCGTTGGAGAATTTC	60
	Zm00014ba353440-DPE2-qR	CCCACCACATCCCATGAACTGAAG	60
Zm00014ba400960-PETE	Zm00014ba400960-PETE-qF	GACCTACTCCGTCACCCTCACC	60
	Zm00014ba400960-PETE-qR	GATCTTGCCGACCATTCCTGCTC	60
Zm*Actin*	Zm*Actin*-qF	CTATCCAGGCTGTTCTTTCGTT	60
	Zm*Actin*-qR	TCAGGCATCTCGTAGCTCTTCT	60

## Data Availability

The data presented in this study are available on request from the corresponding author. Due to privacy and ethical restrictions, the data are not publicly available.
